# Wearable EMI Shielding Composite Films with Integrated Optimization of Electrical Safety, Biosafety and Thermal Safety

**DOI:** 10.1002/advs.202400887

**Published:** 2024-04-19

**Authors:** Liang Li, Yongzhu Yan, Jufu Liang, Jinchuan Zhao, Chaoyi Lyu, Haoxiang Zhai, Xilong Wu, Guizhen Wang

**Affiliations:** ^1^ Center for Advanced Studies in Precision Instruments Center for New Pharmaceutical Development and Testing of Haikou State Key Laboratory of Marine Resource Utilization in South China Sea School of Material Science and Engineering Hainan University Haikou Hainan 570228 China; ^2^ School of Biomedical Engineering State Key Laboratory of Marine Resource Utilization in South China Sea Hainan University Haikou Hainan 570228 China

**Keywords:** biosafety, electrical safety, electromagnetic interference shielding, thermal safety

## Abstract

Biomaterial‐based flexible electromagnetic interference (EMI) shielding composite films are desirable in many applications of wearable electronic devices. However, much research focuses on improving the EMI shielding performance of materials, while optimizing the comprehensive safety of wearable EMI shielding materials has been neglected. Herein, wearable cellulose nanofiber@boron nitride nanosheet/silver nanowire/bacterial cellulose (CNF@BNNS/AgNW/BC) EMI shielding composite films with sandwich structure are fabricated via a simple sequential vacuum filtration method. For the first time, the electrical safety, biosafety, and thermal safety of EMI shielding materials are optimized integratedly. Since both sides of the sandwich structure contain CNF and BC electrical insulation layers, the CNF@BNNS/AgNW/BC composite films exhibit excellent electrical safety. Furthermore, benefiting from the AgNW conductive networks in the middle layer, the CNF@BNNS/AgNW/BC exhibit excellent EMI shielding effectiveness of 49.95 dB and ultra‐fast response Joule heating performance. More importantly, the antibacterial property of AgNW ensures the biosafety of the composite films. Meanwhile, the AgNW and the CNF@BNNS layers synergistically enhance the thermal conductivity of the CNF@BNNS/AgNW/BC composite film, reaching a high value of 8.85 W m^‒1^ K^‒1^, which significantly enhances its thermal safety when used in miniaturized electronic device. This work offers new ideas for fabricating biomaterial‐based EMI shielding composite films with high comprehensive safety.

## Graphical abstract

Wearable electromagnetic interference (EMI) shielding composite films with integrated optimization of electrical safety, biosafety, and thermal safety are fabricated. The composite film also shows ultra‐fast response and long‐term stable Joule heating properties. This work offers new ideas for designing wearable EMI shielding composite films with high comprehensive safety.

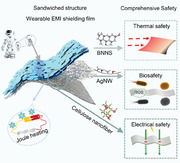

## 1 Introduction

With the widespread use of fifth‐generation wireless and modern communication electronic devices in our daily lives, electromagnetic interference (EMI) pollution has become a growing concern.^[^ ^1^, ^2^, ^3^, ^4^, ^5^, ^6^ ^]^ It could potentially pose detrimental effects on human health, precipitate irreversible malfunctions, and impair the operation of electronic equipment.^[^ ^7^, ^8^, ^9^ ^]^ To effectively combat this problem, a lot of effort has been paid to the development of efficient EMI shielding materials including metal‐type,^[^ ^10^ ^]^ carbon‐type,^[^ ^7^, ^11^ ^]^ MXene‐type,^[^ ^2^, ^3^ ^]^ and conductive polymer composites.^[^ ^6^, ^12^, ^13^ ^]^ Many composite materials in different structural forms, such as compact and laminate structures,^[^ ^14^, ^15^, ^16^, ^17^, ^18^ ^]^ layer‐by‐layer assemblies,^[^ ^19^, ^20^, ^21^ ^]^ porous foams and aerogels,^[^ ^22^, ^23^, ^24^ ^]^ and segregated structures,^[^ ^25^, ^26^ ^]^ have been investigated to enhance the intrinsic EMI shielding property. The latest research indicates that the maximum EMI shielding capability of MXene material can reach 116 dB at a thickness of 40 µm, demonstrating outstanding EMI shielding effectiveness (EMI SE) sufficient to meet the demanding requirements of practical applications.^[^ ^2^ ^]^ However, the comprehensive safety under daily and military environments, which is crucial for the practical application of EMI shielding materials is often neglected.

Specifically, in the field of wearable electronics devices, the following safety issues need to be considered and effectively addressed. First, composite films that exhibit excellent high EMI shielding performance generally show high conductivity, which restricts their application in the smart wearable field.^[^ ^2^ ^]^ The main reason is that excessive conductivity in wearable devices not only has the potential to pose a danger to human health through the risk of current leakage but also threatens the functionality of integrated electronics.^[^ ^27^ ^]^ Ensuring the electrical safety of EMI shielding composite films in smart wearable devices has become an urgent challenge. Secondly, the extensive use of flexible electronic devices in direct contact with human skin increases the potential for cross‐infection. In particular, the prevalence of various infectious diseases, including COVID‐19, along with the recurrence of bacterial infections in chronic patients, will result in significant economic losses and physical pains.^[^ ^28^, ^29^ ^]^ Therefore, biosafety also becomes a pivotal factor for ensuring the safety of EMI shielding composite films. Thirdly, the significance of thermal safety cannot be ignored in the application of integrated smart wearables. With the integration and miniaturization of smart wearable electronic devices, the heat generated by them may not only malfunction or reduce the service life of electronic components but also pose a threat to life safety.^[^ ^30^, ^31^ ^]^ Therefore, it is highly desirable to carry out reasonable structural design for next‐generation EMI shielding materials to solve electrical safety, biosafety, and thermal safety challenges simultaneously.

In this work, well‐designed sandwich‐structured composite films through decorating cellulose nanofibers@boron nitride nanosheet (CNF@BNNS) and silver nanowire (AgNW) on bacterial cellulose (BC) hydrogel (CNF@BNNS/AgNW/BC) were prepared by a simple sequential vacuum filtration method. The CNF@BNNS/AgNW/BC composite films with biocompatible BC hydrogel as the bottom layer, highly conductive AgNW as the middle layer, and high thermal conductivity (TC) of CNF@BNNS as the upper layer demonstrate outstanding EMI SE up to 49.95 dB and achieve integrated optimization of electrical safety, biosafety, and thermal safety. Among them, BC and CNF@BNNS with good electrical insulation separately as the bottom and upper layers of sandwich‐structured CNF@BNNS/AgNW/BC composite films can reduce the possibility of electric shock, thus improving electrical safety. The interconnected AgNW in the middle layer can form an efficient conductive network, providing the CNF@BNNS/AgNW/BC composite films with excellent EMI shielding and ultra‐fast response Joule heating properties. Moreover, benefiting from the intrinsic antibacterial activity of AgNW, the CNF@BNNS/AgNW/BC composite films exhibit excellent antifouling properties, including antibacterial and antibiofilm properties. Thus, when these composite films are applied to shared wearable devices, cross‐infection can be effectively avoided. Meanwhile, CNF@BNNS gives the composite films outstanding thermal management performance, possibly effectively addressing the overheating problem in integrated electronic devices. The CNF@BNNS/AgNW/BC composite films also show outstanding durability performance. They still maintain satisfactory EMI shielding efficiency even when bent repeatedly or treated at high temperatures. Therefore, the CNF@BNNS/AgNW/BC composite films with multiple safety functions considered may have potential applications in shared flexible electronics to prevent cross‐infection and highly integrated electrical devices to enhance their security performance. This work reported here to designing EMI shielding composite films with comprehensive safety considerations may arouse broad interest in developing multifunctional EMI shielding materials to broaden their application scenarios.

## 2 Experimental Section

### 2.1 Materials

Polyvinylpyrrolidone (PVP) and ferric chloride (FeCl_3_) were obtained from Aladdin Reagent Co., Ltd, China. Silver nitrate (AgNO_3_, AR), ethylene glycol (EG, AR), hydrochloric acid (HCl, 37–38 wt.%), and ethanol were purchased from Sinopharm Chemical Reagent Co., Ltd, China. Hexagonal boron nitride (h‐BN) was supplied by Shanghai Naiou Nano Technology Co., Ltd, China. Isopropanol was purchased from China Maclean's Reagent Co. Carbonylated cellulose nanofibers (CNF) aqueous solution (1 wt.%) was obtained from Guilin Qihong Technology Co., Ltd. Bacterial cellulose (BC) hydrogel was purchased from Hainan Yide Food Co., Ltd. The BC hydrogel was purified by immersing them in a 0.1 m solution of sodium hydroxide (NaOH). Following this treatment, the BC hydrogel was thoroughly washed to eliminate any impurities, with the process continuing until the pH of the washing water reached a neutral level of 7. All materials were utilized in their original form and did not undergo any additional purification processes.

### 2.2 Preparation of CNF@BNNS/AgNW/BC Composite Films

**Figure 1:**
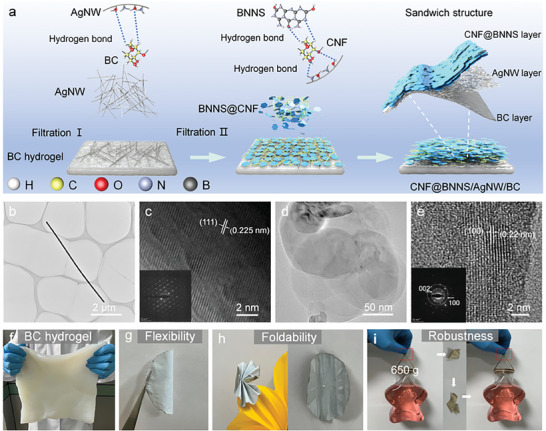
a) Schematic diagram for the preparation of the CNF@BNNS/AgNW/BC composite films. The b) transmission electron microscopy (TEM), c) HRTEM, and selected area electron diffraction (SAED, inset) images of AgNW. The d) TEM, e) HRTEM, and SAED images (inset) of BNNS. f) Digital image of BC hydrogel. Digital images of CNF@BNNS/AgNW/BC composite films for g) flexibility, h) foldability, and i) robustness exhibitions.

The preparation of BNNS and AgNW are described in the Supporting Information.^[^ ^32^, ^33^ ^]^ The sandwich‐structured CNF@BNNS/AgNW/BC composite films were prepared by a simple vacuum‐assisted filtration process, as shown in **Figure** **1**a. BC hydrogel, AgNW aqueous solution (1 mg mL^‒1^), and CNF@BNNS_X_ mixed solution (10 mg mL^‒1^) were respectively filtered successively as the bottom, middle, and top layers of the composite films. Finally, the obtained CNF@BNNS/AgNW/BC composite films were air‐dried at room temperature. For the sake of brevity, CNF@BNNS_x_/5‐AgNW/BC composite films prepared with a different mass of CNF, BNNS, AgNW, and BC were noted as S1 (50, 30, 0, 20 mg), S2 (50, 30, 5, 20 mg), S3 (40, 40, 5, 20 mg), S4 (30, 50, 5, 20 mg), S5 (20, 60, 5, 20 mg), S6 (10, 70, 5, 20 mg), respectively (Table S1, Supporting Information). For comparison, the AgNW aqueous solution was filtered on the surface of BC substrate film to prepare Y‐AgNW/BC films, where Y (1‒5 mg) represented the mass of AgNW.

### 2.3 Characterizations

The detailed characterizations are provided in the Supporting Information.

## 3 Results and Discussion

### 3.1 Characterization of CNF@BNNS/AgNW/BC Composite Films

Figure 1a depicts the synthetic route of sandwich‐structured CNF@BNNS/AgNW/BC composite films. It was worth noting that the top and bottom of the sandwich‐structured composite films consist of CNF@BNNS layer and BC layer, respectively, with AgNW serving as the conductive layer in the middle. As shown in Figure 1b and Figure S1 (Supporting Information) AgNW possesses a high aspect ratio with an average length of 23.78 µm. In addition, the inter‐planar spacing observed in the adjacent lattice fringes of the AgNW is ≈0.225 nm. This measurement correlates well with the expected spacing of the (111) crystallographic planes of the face‐centered cubic (fcc) lattice structure characteristic of silver crystals.^[^ ^34^ ^]^ According to the diffraction ring of AgNW (Figure 1c), its crystal form is a fcc crystal structure, consistent with the (200), (111), (220), and (311) planes in the XRD diffraction peaks.^[^ ^35^ ^]^ A typical TEM image (Figure 1d,e) demonstrates that the BNNS displays an ultrathin 2D lamellar morphology alongside a highly ordered lattice structure. The interlayer distance measured at 0.22 nm is in close agreement with the spacing of the (100) planes of h‐BN. This observation suggests that the crystalline structure of the BNNS has been largely retained.^[^ ^36^ ^]^ As shown in Figure 1f and Figure S2 (Supporting Information), the rich network structure inside the BC hydrogel may provide flexibility to the composite films. The BC and CNF@BNNS layers endow CNF@BNNS/AgNW/BC composite films with excellent flexibility and strength, which allows these composite films to be folded in half (Figure 1g) and folded into complicated shapes without noticeable damage (Figure 1h; Figure S3, Supporting Information). Moreover, the CNF@BNNS/AgNW/BC composite films can withstand a 650 g bottle without breaking even when it is folded at various angles (Figure 1i). These phenomena demonstrate the super flexibility and mechanical properties of CNF@BNNS/AgNW/BC composite films.

**Figure 2:**
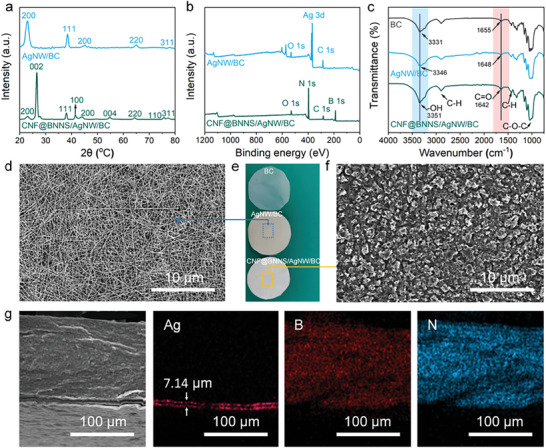
a) XRD patterns, b) XPS spectra, c) FTIR spectra, and d,f) SEM images on the surface of AgNW/BC and CNF@BNNS/AgNW/BC composite films. e) Digital photos of BC, AgNW/BC, and CNF@BNNS/AgNW/BC composite films. g) Elemental maps of B, N, and Ag on the cross‐sectional of CNF@BNNS/AgNW/BC composite films.

The structure and morphology of AgNW/BC and CNF@BNNS/AgNW/BC composite films were investigated by XRD, XPS, FTIR, and SEM. The peak locations and the corresponding crystallographic indices of the AgNW/BC are 22.87° (200), 38.39° (111), 45.10° (200), 64.94° (220), and 78.20° (311), respectively, which are consistent with those reported in ref. [35] (**Figure** **2**a). The CNF@BNNS/AgNW/BC composite films exhibit peaks with crystallographic indices of 22.87° (200), 26.52° (002), 38.50° (111), 41.70° (100), 44.09° (200), 54.67° (004), 64.50° (220), 75.66° (110), and 77.34° (311), respectively, which is consistent with the crystal patterns of AgNW and BNNS.^[^ ^35^ ^]^ XRD patterns and TEM images show that AgNW and BNNS maintain their complete crystalline structure during the preparation process. XPS spectra are shown in Figure 2b. The peaks in the AgNW/BC at binding energies of 285, 533, and 372 eV are attributed to C 1s, O 1s, and Ag 3d, respectively.^[^ ^37^, ^38^ ^]^ This result demonstrates that AgNW was successfully decorated onto BC. On the CNF@BNNS/AgNW/BC composite films, the characteristic C and O peaks location at 285 and 533 eV remain constant, with novel B 1s peak at 189.30 eV and N 1s peak at 395.90 eV.^[^ ^39^ ^]^ Since XPS is being used to detect the elemental composition of the surface species, the characteristic Ag 3d peak wasn't detected in the XPS spectra of the CNF@BNNS/AgNW/BC composite films. This further indicates that the sandwich structure of the CNF@BNNS/AgNW/BC composite films was successfully fabricated. In addition, the strong interfacial interaction (hydrogen bonding) between the AgNW and BC as well as CNF were confirmed by FTIR spectra, as shown in Figure 2c. Comparing the FTIR spectra of BC, AgNW/BC, and CNF@BNNS/AgNW/BC composite films, the hydroxyl peak at 3339 cm^‒1^ and the carboxyl peak at 1645 cm^‒1^ in pure BC, which were shifted to lower wavenumbers during composite films formation. The movement of these peaks confirms the hydrogen bonding between the hydroxyl group of AgNW and the hydroxy carboxyl group on BC and CNF. This hydrogen bonding effect increases the interlayer bonding force of the sandwich‐structured composite films, improving its flexibility and mechanical strength.^[^ ^19^, ^21^, ^31^ ^]^ From Figure 2d–f, the one‐dimensional AgNW and two‐dimensional BNNS were uniformly, densely, and oriented dispersed, and the shape of the composite films is regular (circular), with silver gray color on the upper surface of AgNW/BC and white color on the upper surface of CNF@BNNS/AgNW/BC composite films. The elemental maps of the CNF@BNNS/AgNW/BC composite films show the sandwich structure, that is, B and N are integrated into the upper layer and Ag is integrated into the middle layer. Moreover, the thickness of the AgNW interlayer is only 7.14 µm, which is very meaningful for constructing efficient electron and phonon conduction paths (Figure 2g). Such a sandwich structure for CNF@BNNS/AgNW/BC composite films is expected to simultaneously solve the electrical safety, biosafety, and thermal safety of the composite films due to its electrical insulation property of the surface, antibacterial property of AgNW and excellent TC of BNNS.

### 3.2 Sheet Resistance and EMI Shielding Performance of CNF@BNNS/AgNW/BC Composite Films

**Figure 3:**
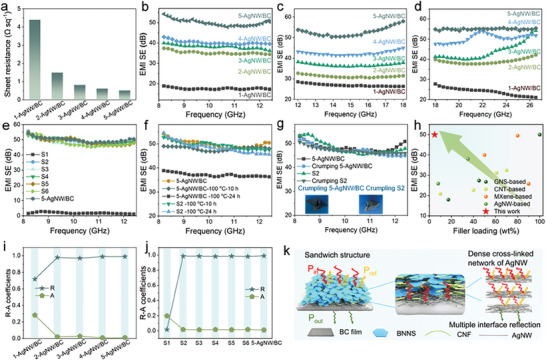
a) Sheet resistance, EMI shielding performance in b) X‐band, c) Ku‐band, and d) K‐band of AgNW/BC composite films. e) The EMI shielding performance of CNF@BNNS_30_/BC, CNF@BNNS/AgNW/BC, and 5‐AgNW/BC composite films. f) The EMI shielding performance of 5‐AgNW/BC, and CNF@BNNS/AgNW/BC composite films at 100 °C for 10 and 24 h. g) The EMI shielding performance of the 5‐AgNW/BC, CNF@BNNS/AgNW/BC, crumpling 5‐AgNW/BC, and CNF@BNNS/AgNW/BC composite films. h) Comprehensive comparison of EMI SE of CNF@BNNS/AgNW/BC composite films with other reported composite films. Corresponding power coefficients of reflectivity (*R*) and absorptivity (*A*) values in X‐band of i) AgNW/BC composite films, j) CNF@BNNS_30_/BC, CNF@BNNS/AgNW/BC, and 5‐AgNW/BC composite films. k) EMI shielding mechanism of CNF@BNNS/AgNW/BC composite films.

Electrical conductivity is a pivotal factor influencing the EMI shielding performance of composite films.^[^ ^3^, ^14^, ^40^ ^]^ **Figure** **3**a illustrates the sheet resistance (*R*_S_) of AgNW/BC composite films. AgNW/BC composite films show a monotonous decrement in *R*_S_ with increasing the mass of AgNW. In particular, the *R*_S_ of 5‐AgNW/BC composite film is 0.52 Ω sq^‒1^, due to the dense conductive path (Figure S4, Supporting Information). The EMI shielding performance of the AgNW/BC composite films is obtained according to Equations S1−S5 (Supporting Information) (Figure 3b−d). Pure BC film exhibits weak EMI shielding performance due to its electrical insulation (Figure S5, Supporting Information). Compared with BC film, benefiting from the low *R*_S_, the EMI shielding property of the AgNW/BC composite films exceeds the standards of commercial EMI shielding materials (EMI SE>10 dB). Meanwhile, the AgNW/BC composite films both reveal outstanding EMI SE in the Ku‐band and K‐band (Figure 3c,d). It can be seen in Figure 3b−d, that AgNW/BC composite films exhibit significantly increased total EMI shielding performance from 17.64 to 49.95 dB with increased content of AgNW. As shown in Figure S6 (Supporting Information), EMI shielding efficiencies of AgNW/BC composite films increased from 98.274% to ≈99.999% with the increase of the AgNW conduction path (Equation S5, Supporting Information). Continuously increasing the mass of AgNW does not enhance the EMI SE of the AgNW/BC composite films (Figure S7, Supporting Information), owing to the dense conductive network was fully established when 5 mg of AgNW was added (Table S2, Supporting Information). Consequently, a quantity of 5 mg of AgNW was selected to produce the sandwich‐structured composite films.

The addition of the CNF@BNNS layer does not disrupt the integrated and dense electromagnetic wave (EMW) reflection path formed by AgNW, thus the CNF@BNNS/AgNW/BC composite films still have satisfactory EMI shielding performance (Figure 3e; Figure S8, Supporting Information). The CNF@BNNS and BC layers also confer electrical insulation to the external surfaces of the CNF@BNNS/AgNW/BC composite films, thereby the structural design of the sandwich is necessary to address the electrical safety of the EMI shielding composite films. More importantly, the EMI shielding performance of the CNF@BNNS/AgNW/BC composite films was not degraded compared to the AgNW/BC film after 10 and 24 h of treatment at 100 °C, which is attributed to the unique sandwich structure that protects the AgNW in the middle layer from oxidation (Figure 3f). Furthermore, the EMI shielding performance of the CNF@BNNS/AgNW/BC composite films has not deteriorated seriously after being wrinkled, this stable EMI shielding performance increases its application in the smart wearable field (Figure 3g; Figure S9, Supporting Information). As shown in Figure 3h, Tables S3 and S4 (Supporting Information), CNF@BNNS/AgNW/BC composite film presents an excellent EMI SE of 49.95 dB at AgNW additions of 4.76 wt.%, which exceeds many previously reported EMI shielding films with the same amount of filler.^[^ ^19^, ^23^, ^30^, ^41^, ^42^, ^43^, ^44^, ^45^, ^46^, ^47^, ^48^, ^49^ ^]^

In pursuit of exploring the EMI shielding mechanism, the average SE_R_, SE_A_, SE_T,_ and *R* and *A* coefficients are calculated according to Equations S1−S4 (Supporting Information) (Figure S10, Supporting Information; Figure 3i,j). As shown in Figure S10 (Supporting Information), the SE_A_ values of AgNW/BC and CNF@BNNS/AgNW/BC composite films are remarkably greater than the corresponding SE_R_ values. However, most of the EMW is first reflected when it reaches the AgNW layer due to the impedance mismatch between space or CNF@BNNS layer and conductor, and the remaining EMW is absorbed inside the material by dielectric and conduction losses. Thus, AgNW/BC and CNF@BNNS/AgNW/BC composite films with SE_R_ values >10 dB imply that they reflected >90% of the EMW that reached the AgNW layer. *R* and *A* coefficients are used to evaluate the interaction between EMW and AgNW/BC and CNF@BNNS/AgNW/BC composite films (Figure 3i,j). The *R* coefficients of both AgNW/BC and CNF@BNNS/AgNW/BC composite films are much larger than the *A* coefficients, indicating that reflection is the dominant mechanism for EMI shielding. To understand the EMI shielding mechanism of the sandwich‐structured CNF@BNNS/AgNW/BC composite films, an EMW transmission mechanism diagram was given in Figure 3k. First, the EMW all passes through the electrically insulating CNF@BNNS layer, when the EMW reaches the AgNW layer, due to an impedance mismatch, >90% of the incident EMW is reflected immediately when they come into contact with the AgNW.^[^ ^50^ ^]^ Then, the incident EMW is reflected several times in the dense AgNW conductive layer. During this process, EMW interacted with the high electron density of AgNW, generating a current that causes ohmic losses and polarization loss, resulting in the energy of EMW drop being converted into heat energy. The rich interface formed by the integrated AgNW direct cross–linking facilitates repeated reflection of residual EMW until they are consumed, resulting in lower EMW transmittance. In summary, the combination of the low R_S_ of AgNW, the integrated and dense oriented structure, and multiple‐conducting cross–linked networks achieve excellent EMI shielding performance.

### 3.3 Biosafety of CNF@BNNS/AgNW/BC Composite Films

**Figure 4:**
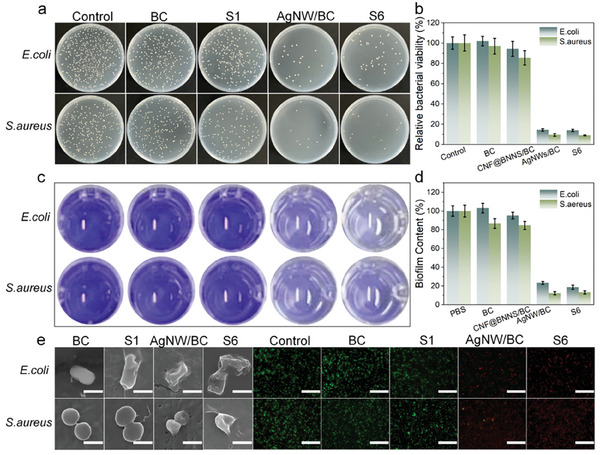
Antibacterial and antibiofilm tests of different samples in *E. coil* and *S. aureus* bacterial suspensions. a) Images and b) size statistics of bacterial viability. Crystal violet staining of samples in *E. coil* and *S. aureus* bacterial suspensions c) images and d) biofilm content. e) SEM images and live/dead double fluorescence staining of bacteria adhered on the surfaces of composite films (scale bar represents 1 µm).

Biosafety (antibiofouling performance) is crucial for the practical application of smart wearable composite films. To investigate the antibacterial efficiency of composite films, the inhibitory effects of composite films were examined against gram‐negative *E. coli* and gram‐positive *S. aureus*. The antibacterial activities of the samples were tested by diffusion tests in agar plates according to the dilution coating plate method (Figure S11, Supporting Information; **Figure** **4**a,b). The control group exhibited a massive number of bacterial colonies. As compared, the AgNW/BC and CNF@BNNS/AgNW/BC composite films both exhibit excellent antibacterial capability against the two types of bacteria, which could be demonstrated by the large diameter of the inhibitory zone and the lower relative bacterial viability (Equation S6, Supporting Information). In addition, excellent antibiofilm capability is very necessary for wearable materials owing to bacterial biofilms that can lead to repeated bacterial infection of human wounds. Crystal violet staining was used to assess the formation and destruction of biofilm. The dark blue color of control, BC, and CNF@BNNS/BC groups indicates the formation of biofilms (Figure 4c). However, the AgNW/BC and CNF@BNNS/AgNW/BC treatment groups present a light blue color, demonstrating that the biofilm was obviously damaged.^[^ ^51^ ^]^ Quantitative analysis of biofilm mass by measuring optical density at 570 nm (Figure 4d).^[^ ^52^ ^]^ The biofilm content on the AgNW/BC and CNF@BNNS/AgNW/BC composite films was reduced by ≈80% compared to that on the control group, BC, and CNF@BNNS/BC films. These results suggest that AgNW/BC and CNF@BNNS/AgNW/BC composite films show antibiofilm activity, owing to Ag^+^‐induced membrane damage and bacterial inactivation.^[^ ^53^, ^54^ ^]^

In addition, the bacteria attached to the different composite films were observed using the SEM and fluorescent microscope after 48 h of treatments. Bacteria on the cell membranes of BC and CNF@BNNS/BC films are intact and smooth. In contrast, SEM images show shrunken cell membranes in AgNW/BC and CNF@BNNS/AgNW/BC composite films, which is attributed to the leakage of intracellular material from the damaged bacteria (Figure 4e). Cell membrane damage was further assessed by Bac Light staining for live/dead bacterial activity. Green fluorescent SYTO 9 can quickly enter any cell wall and bind to DNA, while red‐fluorescent propidium iodide only enters cells with damaged plasma membranes.^[^ ^51^ ^]^ No red spots were found in control, BC, and CNF@BNNS/BC, demonstrating that the bactericidal capacity of these films is weak. Many red spots in the AgNW/BC and CNF@BNNS/AgNW/BC composite films are further evidence of their high activity inhibiting bacterial contamination (Figure 4e). The excellent antibacterial and antibiofilm properties of the AgNW/BC and CNF@BNNS/AgNW/BC composite films are mainly due to the fact that Ag^+^ converts water into hydrogen peroxide in the catalytic medium in the presence of atmospheric oxygen, and then converts into reactive oxygen species, which further oxidizes the molecular structure of bacteria, thus inhibiting the growth of surrounding bacteria.^[^ ^55^ ^]^ In this work, the CNF@BNNS/AgNW/BC composite films exhibit outstanding antibacterial and antibiofilm capabilities, which is a promising wearable material with satisfactory biosafety.

### 3.4 Thermal Safety of CNF@BNNS/AgNW/BC Composite Films

**Figure 5:**
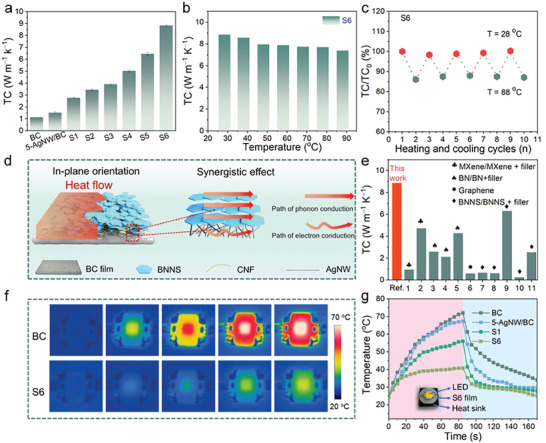
a) In‐plane TC of BC, AgNW/BC, CNF@BNNS/BC, and CNF@BNNS/AgNW/BC composite films. b) Temperature stability and c) cycle thermal stability of the CNF@BNNS_70_/5‐AgNW/BC composite film. d) Scheme showing the mechanism of thermal conductivity of CNF@BNNS/AgNW/BC composite films. e) Comparison of TC between CNF@BNNS/AgNW/BC composite film with composite films reported in the literature. f) Surface thermal imagery of practical demo monitored by the thermal infrared imager. g) The corresponding temperature of the test points as a function of time, inset is the photo of the LED chip integrated with composite films and heat sink.

For portable electronic products, the integrated electronic components therein generate excessive thermal energy, which threatens their function, service life, and safety; thus, EMI shielding composite films with satisfactory thermal safety are necessary for such electronic products.^[^ ^14^, ^30^ ^]^ **Figure** **5**a shows the TC performance of BC, AgNW/BC, CNF@BNNS/BC, and CNF@BNNS/AgNW/BC composite films (Equation S7, Supporting Information). The in‐plane TC of the pure BC film at 30 °C is ≈1.14 W m^−1^∙K^−1^. The CNF@BNNS/AgNW/BC composite films show a higher TC due to the high orientation of AgNW and BNNS along the in‐plane direction. It can be observed that the in‐plane TC of the CNF@BNNS/AgNW/BC composite films can effectively improve by increasing the AgNW and BNNS loading. The TCs of CNF@BNNS_30_/BC and CNF@BNNS_30_/5‐AgNW/BC composite films are 2.77 and 3.44 W m^−1^ K^−1^, respectively, indicating that AgNW and BNNS synergically increase the TC of composite films. When the BNNS content is 70 mg, the TC increases to 8.85 W m^−1^ K^−1^. The CNF@BNNS layer not only prevents the oxidation of AgNW but also endows excellent thermal management properties to the CNF@BNNS/AgNW/BC composite films. To demonstrate its practical application in high temperature‐resistant wearable devices, the TCs of CNF@BNNS_70_/5‐AgNW/BC composite film at different temperatures and temperature cycling were performed. Figure 5b shows that the CNF@BNNS_70_/5‐AgNW/BC composite film maintains high TC at high temperatures. Figure 5c reveals that the TC of the CNF@BNNS_70_/5‐AgNW/BC composite film remains stable when tested for 10 cycles within the range of 28−88 °C, with the maximum TC errors of 1.68% and 1.65% at 28 and 88 °C, respectively.^[^ ^56^, ^57^ ^]^

BNNS were exfoliated while preserving the crystalline lattice integrity, manifesting a large aspect ratio that facilitates inter‐nanosheet overlap. During the vacuum‐assisted filtration process, the well‐dispersed BNNS is highly stacked in the CNF@BNNS layer along the in‐plane direction, forming an internal interconnected phonon conduction network. The integrated orientation of AgNW also increases the electron conduction path. The integrated phonon and electron transport paths formed by the in‐plane orientation of BNNS and AgNW work synergistically to improve the thermal management performance of CNF@BNNS/AgNW/BC composite films (Figure 5d). Figure 5d shows a possible thermal conduction model for the CNF@BNNS/AgNW/BC composite films. In addition, it is noteworthy that the in‐plane TC of the CNF@BNNS/AgNW/BC composite films is better than similar material as previously reported, which fulfills the demands of thermal management devices. (Figure 5e; Table S5, Supporting Information).^[^ ^58^, ^59^, ^60^, ^61^, ^62^, ^63^, ^64^, ^65^, ^66^, ^67^, ^68^, ^69^ ^]^

Moreover, the real heat diffusion process in the center of the composite films heated by the laser beam (808 nm) was recorded by the IR camera (Figure S12, Supporting Information). To avoid the phenomenon of light transmission through the composite films, both sides of all composite films were sprayed with graphite. The CNF@BNNS_70_/5‐AgNW/BC composite film with a high TC shows a faster heat diffusion rate and higher steady‐state saturation temperature (*Ts*). For instance, the center of the CNF@BNNS_70_/5‐AgNW/BC composite film can reach a Ts of up to 74 °C due to rapid heat diffusion, while the center of 5‐AgNW/BC composite film can reach a much lower Ts of just 37.8 °C due to a slower thermal diffusion rate. Hence, the CNF@BNNS_70_/5‐AgNW/BC composite film presents great advantages in practical thermal management applications. Furthermore, the CNF@BNNS_70_/5‐AgNW/BC composite film has important applications as TIM for electronic devices. The AgNW/BC, CNF@BNNS/BC, and CNF@BNNS/AgNW/BC TIM were used between light‐emitting diodes (LED) and heat sinks, and an IR camera was utilized to dynamically monitor the surface temperature of the LED. For comparison, BC was also used in TIM. When a BC film was used as the TIM, the surface temperature of the LED chip increased significantly; in fact, its highest operating temperature exceeded 70.1 °C (Figure 5f,g). However, the LED's temperature showed a lower *T*s with 40.5 °C when CNF@BNNS_70_/5‐AgNW/BC composite film was used as the TIM. The reduced surface temperature of the LED chip is evidence that CNF@BNNS/AgNW/BC composite films with the oriented BNNS and AgNW network structure are very effective at dissipating heat energy. Therefore, the sandwich‐structured CNF@BNNS/AgNW/BC composite films demonstrate huge potential in practical thermal management applications.

### 3.5 Joule Heating Performance of CNF@BNNS/AgNW/BC Composite Films

**Figure 6:**
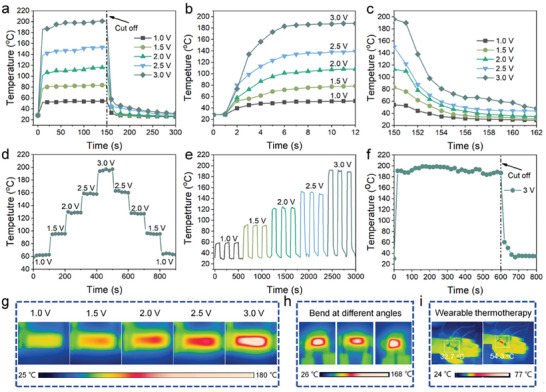
a–c) Surface temperature variations of CNF@BNNS_70_/5‐AgNW/BC film with different voltages. d) Saturation temperature variation curves of CNF@BNNS_70_/5‐AgNW/BC composite film during voltage adjustment. e) Thermal response of CNF@BNNS_70_/5‐AgNW/BC composite film with different voltages. f) Time‐temperature curve of CNF@BNNS_70_/5‐AgNW/BC composite film at 3 V constant voltage. The infrared images of CNF@BNNS_70_/5‐AgNW/BC composite film at g) switching voltage, h) bending at different angles at 3 V. i) IR camera images of the electrical heaters in wearable thermotherapy.

The flexible electronic devices with Joule heating function can be used for defogging and dehumidification, which gives them great potential to apply under extreme environments. Therefore, the Joule heating effect of the composite films was investigated. Due to a good double‐sided insulation feature, the flexible CNF@BNNS/AgNW/BC composite films can serve as an electric heater with electrical safety by utilizing the Joule heating effect for electricity‐heat conversation. When the electric current passes through the CNF@BNNS_70_/5‐AgNW/BC composite film at the supplied voltage, heat energy is produced because of the inelastic collision between the accelerated electron and phonon.^[^ ^37^ ^]^ **Figure** **6**a shows the time‐dependent surface temperature of electrical heaters at low supplied voltages from 1 to 3 V. Driven by the supplied voltage, the surface temperature of electrical heaters rapidly increases due to the generation of Joule heat and swiftly reaches the *T*s. The increased voltage can accelerate the collision of electrons with positive ions, so the *T*s of the CNF@BNNS_70_/5‐AgNW/BC composite film increases with increasing voltage. Among them, the *T*s of the CNF@BNNS_70_/5‐AgNW/BC composite film is up to 190 °C when supplied with a voltage of 3 V. In addition to ultra‐high *T*s, response time is another factor that should be considered in the practical application of materials with Joule heating. After the supplied voltage is switched off, the temperature of the electrical heater immediately drops and the working state quickly stops. Notably, the CNF@BNNS_70_/5‐AgNW/BC composite film has an ultra‐fast response Joule heating performance, which can achieve rapid rise and fall of *T*s within 8 s. (Figure 6a−c). This can be attributed to the integrated conductive pathway of AgNW. In addition, temperature response and temperature stability are equally important for the application of composite films with Joule heating. Figure 6d exhibits the temperature response of CNF@BNNS_70_/5‐AgNW/BC composite film at different input voltages. The temperature response of CNF@BNNS_70_/5‐AgNW/BC composite film is rapid without obvious hysteresis when there is a gradient increase or decrease in input voltage, which indicates that CNF@BNNS_70_/5‐AgNW/BC composite film has great potential for development in the field of intelligent temperature control.^[^ ^34^ ^]^ Figure 6e presents that the *T*s of CNF@BNNS_70_/5‐AgNW/BC composite film remains stable during the cold and thermal cycling experiments. It can be seen in Figure 6f that the long‐term heating performance of CNF@BNNS_70_/5‐AgNW/BC composite film is at a constant voltage of 3 V. The CNF@BNNS_70_/5‐AgNW/BC composite film exhibits a remarkably stable heating temperature of ≈190 °C for longer than 10 min, showing their excellent heating stability and reliability for practical applications.

An IR camera was used to evaluate the potential of CNF@BNNS/AgNW/BC composite films with Joule heating for practical applications. The infrared thermal images at different supply voltages in Figure 6g show that the *T*s of the CNF@BNNS_70_/5‐AgNW/BC composite film increase as the voltage increases. In addition, the CNF@BNNS_70_/5‐AgNW/BC composite film possesses satisfactory Joule heating performance even when bent at different angles (Figure 6h). CNF@BNNS_70_/5‐AgNW/BC composite film can be used as a wearable thermal therapy device (Figure 6i). When the CNF@BNNS_70_/5‐AgNW/BC composite film was placed on the hand as an electric heater, the temperature of the electric heater was only 32.7 °C when no voltage was provided, while the temperature of the electric heater increased to 54.3 °C when a voltage of 0.9 V is applied. Thus, the CNF@BNNS/AgNW/BC composite film displays great potential as a stable antimicrobial wearable thermal therapy device for surgical patients in extremely cold regions.

**Figure 7:**
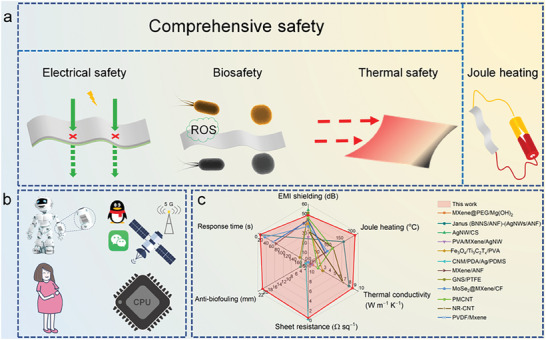
a) The schematic diagram of the comprehensive safety and joule heating performance, and b) application of CNF@BNNS/AgNW/BC composite films. These applications of c) The comprehensive performance comparison between the CNF@BNNS/AgNW/BC composite film and samples reported in the literature.

Based on the above analysis, the sandwich structure simultaneously optimizes the electrical safety, biosafety, and thermal safety of the CNF@BNNS/AgNW/BC composite films, and endows it with excellent Joule heating properties, which makes it have outstanding application potential in many fields such as artificial intelligence, TIM, wearable thermal therapy devices, wearable electronic devices, antibacterial and antiradiation clothing (**Figure** **7**a,b). In addition, surface‐insulating and flexible CNF@BNNS/AgNW/BC composite films with excellent overall performance, including EMI shielding, antibiofouling, thermal management, and Joule heating have obvious advantages compared to many materials reported in the literature (Figure 7c; Table S6, Supporting Information).^[^ ^12^, ^28^, ^35^, ^47^, ^50^, ^58^, ^70^, ^71^, ^72^, ^73^, ^74^, ^75^, ^76^, ^77^ ^]^

## 4 Conclusion

In summary, we demonstrated a facile vacuum‐assisted filtration process for fabricating sandwich‐structured CNF@BNNS/AgNW/BC composite films with outstanding EMI shielding capacity and integrated optimization of electrical safety (surface electrical insulation), biosafety (antibiofouling), and thermal safety (thermal management performance). By employing the sandwiched structure design, the electrical insulation on both the upper and bottom surfaces of the CNF@BNNS/AgNW/BC composite film is guaranteed, ensuring its electrical safety during the application process. The AgNW in the middle layer forms an efficient 3D conductive network on the BC film. With an ultra‐low AgNW content of 4.76 wt.%, the obtained CNF@BNNS/AgNW/BC composite film possesses an outstanding EMI SE of 49.95 dB. In addition, the dense conductive network enables the CNF@BNNS/AgNW/BC composite films to possess ultra‐fast response Joule heating performance, achieving a heating temperature of up to 196 °C at a 3 V supply voltage and rapid heating and cooling cycles within just 8 s. Moreover, the AgNW layer also provides excellent antibacterial and antibiofilm abilities of CNF@BNNS/AgNW/BC composite films. More importantly, the CNF@BNNS on the upper layer of composite films not only protects the AgNW from oxidation but also increases the thermal management capability of the CNF@BNNS/AgNW/BC composite films. The high orientation of BNNS and AgNW endows the CNF@BNNS/AgNW/BC composite film with a high TC of 8.86 W m^‒1^ k^‒1^ and good thermal cycle stability. Accordingly, we believe that the CNF@BNNS/AgNW/BC composite films have promising potential in the field of 5G wearable electronic devices, and the strategy proposed in this study may provide guidance for designing wearable EMI shielding composite films with high comprehensive safety.

## Conflict of Interest

The authors declare no conflict of interest.

## Supporting information

Supporting Information

## Data Availability

The data that support the findings of this study are available from the corresponding author upon reasonable request.
