# Supplementary material for: Wearable EMI Shielding Composite Films with Integrated Optimization of Electrical Safety, Biosafety and Thermal Safety
Source: Adv Sci (Weinh). 2024 Apr 19;11(21):2400887. doi: 10.1002/advs.202400887 (PMC11151081; doi:10.1002/advs.202400887)
Supplement: Supplementary file 1 — Supporting Information [file ADVS-11-2400887-s001.pdf]

ADVANCED SCIENCE
Open Access

Supporting Information

for *Adv. Sci.*, DOI 10.1002/advs.202400887

Wearable EMI Shielding Composite Films with Integrated Optimization of Electrical Safety, Biosafety and Thermal Safety

Liang Li, Yongzhu Yan, Jufu Liang, Jinchuan Zhao, Chaoyi Lyu, Haoxiang Zhai, Xilong Wu* and Guizhen Wang**

Supporting Information

Wearable EMI Shielding Composite Films with Integrated Optimization of Electrical Safety, Biosafety and Thermal Safety

Liang Li, Yongzhu Yan, Jufu Liang, Jinchuan Zhao, Chaoyi Lyu, Haoxiang Zhai, Xilong Wu,* Guizhen Wang**

L. Li, Y. Yan, J. Liang, J. Zhao, H. Zhai, G. Wang
Center for Advanced Studies in Precision Instruments
Center for New Pharmaceutical Development and Testing of Haikou
State Key Laboratory of Marine Resource Utilization in South China Sea, School of Material Science and Engineering
Hainan University
Haikou, Hainan 570228, China
Email: yzyancam@hainanu.edu.cn, wangguizhen@hainanu.edu.cn

C. Lyu, X. Wu
School of Biomedical Engineering, State Key Laboratory of Marine Resource Utilization in South China Sea
Hainan University
Haikou, Hainan 570228, China
Email: wuxilong@hainanu.edu.cn

Experimental section

Preparation of AgNW

Silver nanowire (AgNW) was synthesized via a solution-phase polyol method. Briefly, 0.05 g of PVP (K−30), 0.10 g of PVP (K−90), and $FeCl_3$/EG solution (600 μM, 2.50 mL) were dissolved in 22 mL of EG in a flask and then heated to 140 °C under magnetic stirring. Subsequently, 3 mL of $AgNO_3$/EG solution (60 mg mL^{-1}) was added dropwise to the mixture under stirring for another 120 min. After the completion of the reaction, the solution was cooled in the ambient environment and then washed with ethanol to free PVP and EG. A final dispersion of AgNW in deionized water was obtained.

Preparation of BNNS

About 3.50 g of boron nitride powders were added into a 200 mL mixture solvent of isopropanol and deionized water (1:1 by weight). The solution was sonicated for 4 h through an Ultrasonic machine. The resulting dispersions were centrifuged using a centrifuge at 4000 rpm for 10 min to remove non-exfoliated h-BN. The supernatants were collected and centrifuged at 9000 rpm for 30 min to collect the exfoliated BNNS.

Characterization

The morphologies of BNNS and AgNW were characterized by transmission electron microscopy (TEM, Thermo Scientific, Talos F200X G2), selected area electron diffraction (SAED), scanning electron microscopy (SEM, Thermo Scientific, Verios G4 UC). The morphologies of AgNW/BC, CNF@BNNS/BC, and CNF@BNNS/AgNW/BC composite films were characterized by SEM (Thermo Scientific, Verios G4 UC), equipped with an energy dispersive spectrometer (EDS). X-ray diffraction instruments (XRD, Smart Lab Ⅱ, Cu Kα radiation), X-ray photoelectron spectroscopy (XPS, AXIS SUPRA, Al Kα source) and Fourier transform infrared spectrometer (FTIR, NICOLET 6700) with wavenumber range of 4500 – 650 cm^{-1} was employed to record the element, crystal structure and intermolecular forces of the AgNW/BC, CNF@BNNS/BC, and CNF@BNNS/AgNW/BC composite films.

The Sheet Resistance, EMI shielding, Anti-biofouling, Thermal Conductivity, and Joule Heating Performance Measurements

An RTS-8 four-point probe tester examined sheet resistance (Rs) at the temperature of 25 ºC according to the modified ASTM F 390-98. Electromagnetic parameters were obtained using a network analyzer (Ceyear, 3672B-S) in the frequency of 8.2–12.4 GHz

(X–band: 22.86×10.16 mm), 12.4–18 GHz (Ku–band: 15.86×7.92 mm), 18–26 GHz (K–band: 10.68×4.30 mm). The scattering parameters (S11 and S21) in the X–band, Ku–band, and K–band were recorded to calculate the coefficients of reflection (R), absorption (A) and transmission (T), total EMI SE (SE_T), absorption (SE_A), and reflection (SE_R) using the following equations:

$$R = |S_{11}|^2, T = |S_{21}|^2 \quad \text{(S1)}$$

$$A = 1 - R - T \quad \text{(S2)}$$

$$SE_R = -10log(1-R), SE_A = -10\,log\left(\frac{T}{1-R}\right) \quad \text{(S3)}$$

$$SE_T = SE_R + SE_A + SE_M \quad \text{(S4)}$$

$$Shielding\ efficienty\ (\%) = 100 - \left(\frac{1}{10}\right)^{\frac{SE}{10}} * 100 \quad \text{(S5)}$$

Among them, when SE_T > 15 dB, SEM can be usually ignored.

The anti-biofouling measurements were carried out with two model bacteria (gram-negative E. coli and gram-positive S. aureus). For antimicrobial evaluation, various single colonies were selected in 5 mL Luria-Bertani (LB) liquid medium and incubated at 180 rpm and 37 ºC for 8−10 h to reach the logarithmic growth phase. Suitable concentrations were obtained by diluting the bacterial solutions with phosphate buffer (PBS, pH 7.4). All materials were dried and disinfected with UV irradiation for 60 min before use. The bacterial inhibitory capacity of the material was evaluated using the plate method. All experimental specimens (67 μg mL^{-1}) were co-cultured with bacteria (approximately 3×106 CFU mL^{-1} each). To prevent the effect of heat and light on the test results, the ice packs were changed every 10 min. After 40 min of light, the bacterial solution was diluted and inoculated onto suitable agar and incubated at 37 ºC for 12−14 h. The results were expressed as the total number of colony-forming units (CFU). Bacterial inhibition was quantitatively evaluated by measuring the optical density (OD) and the diameter of the inhibition circle of the nearby bacterial solution at 600 n.

$$relative\ bacterial\ viability\ (\%) = \frac{CFU_0(cell) - CFU(cell+gel+rad)}{CFU_0(cell)} \times 100\% \quad \text{(S6)}$$

The aforementioned composite films (3 mg well^{-1}) were added into each well of a 48-well plate. The previous strains during the logarithmic growth period were diluted 10-fold in sterile saline (3×108 CFU mL^{-1}). 0.30 mL of the above bacterial solution was added to each well and cultured at 37 ˚C for 48 h to form biofilms. The integrity of bacterial biofilms[1] was evaluated by using the LIVE/DEAD BacLight Bacterial

Viability Kit (Invitrogen, USA) and detecting the OD_{260} value of bacterial supernatant, which originated from the released DNA and RNA from the lysed cell. A widely used crystal violet staining assay was used for biofilm quantification.[2] In brief, the biofilms were gently washed with PBS to remove suspended bacteria. Then 0.1 mL of 4% paraformaldehyde was added into each well and kept for 10 min. After removing paraformaldehyde, each sample was stained with 0.10 mL of crystal violet dye for 15 min, and then rinsed in PBS. Afterward, 0.30 mL of absolute ethanol was added to release bound crystal violet. The color of each well was recorded by the camera. The absorbance quantification of the biofilms in each well was measured using a microplate reader at 570 nm. The changes in bacterial biofilms were further identified through SEM. The mats containing bacteria were collected and fixed in glutaraldehyde. Subsequently, the samples were dehydrated gradually with a suite of ethanol–water mixtures, dried under vacuum until constant weight and observed through FE-SEM.

Thermal diffusivity (α) was performed on a NETZSCH LFA 467 Nano Flash at 25 °C. The thermal conductivity (λ, W m^{-1} K^{-1}) was calculated by the following equations, density (ρ, g cm^{-3}), specific heat (Cp, J g^{-1} K^{-1}), and thermal diffusivity (α, mm^2 s^{-1}).

$$\lambda = \alpha \times \rho \times C_p \qquad \text{(S7)}$$

The temperature changes of the center point of the composite films were monitored with a thermal imaging camera (E6, FLIR).

Joule heating performance was characterized by charging at a certain voltage supplied by a DC power supply (UTP 1306S, UNI-T), and the changes in surface temperature were monitored simultaneously by using a thermal imaging camera (E6, FLIR).

Table S1. The specific content of 5-AgNW/BC, CNF@BNNS/BC, and CNF@BNNS/AgNW/BC composite films.

Samples	CNF (mg)	BNNS (mg)	AgNW (mg)	BC (mg)
5-AgNW/BC	0	0	5	20
S1 (CNF_{50}@$BNNS_{30}$/CNF)	50	30	0	20
S2 (CNF_{50}@$BNNS_{30}$/5-AgNW/BC)	50	30	5	20
S3 (CNF_{40}@$BNNS_{40}$/5-AgNW/BC)	40	40	5	20
S4 (CNF_{30}@$BNNS_{50}$/5-AgNW/BC)	30	50	5	20
S5 (CNF_{20}@$BNNS_{60}$/5-AgNW/BC)	20	60	5	20
S6 (CNF_{10}@$BNNS_{70}$/5-AgNW/BC)	10	70	5	20

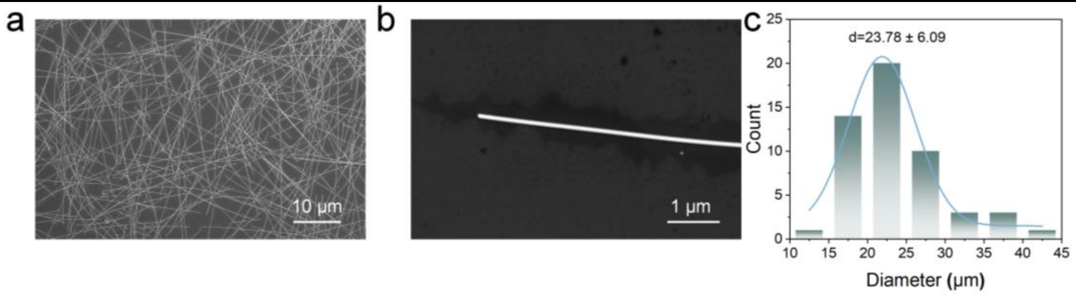

Figure S1. (a), (b) SEM micrographs and (c) radial size statistics of AgNW.

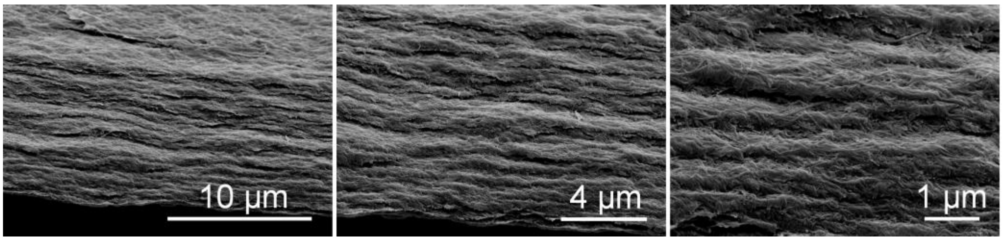

Figure S2. The cross-sectional SEM micrographs of BC film.

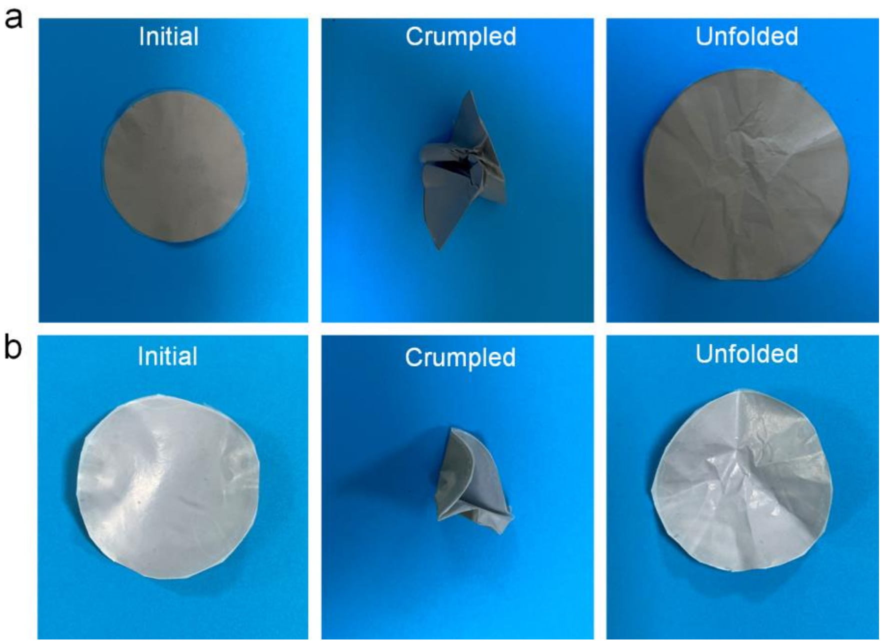

Figure S3. The crumpling process of (a) AgNW/BC and (b) CNF@BNNS/AgNW/BC composite films.

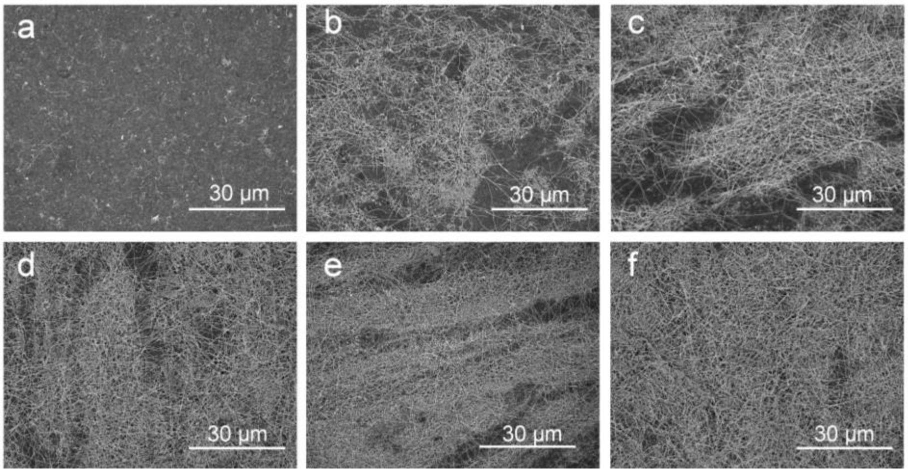

Figure S4. SEM images of the upper surface of (a) BC, (b) 1-AgNW/BC, (c) 2-AgNW/BC, (d) 3-AgNW/BC, (e) 4-AgNW/BC, and (f) 5-AgNW/BC composite film.

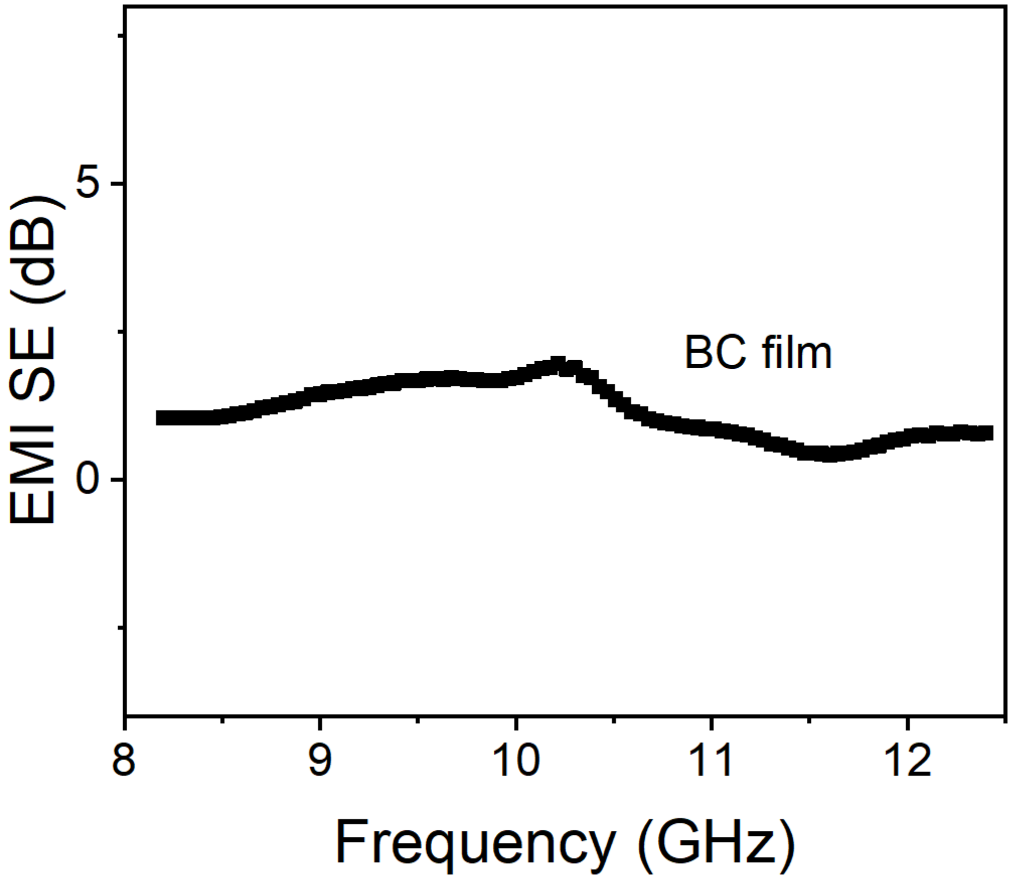

Figure S5. The EMI SE of BC film in X-band.

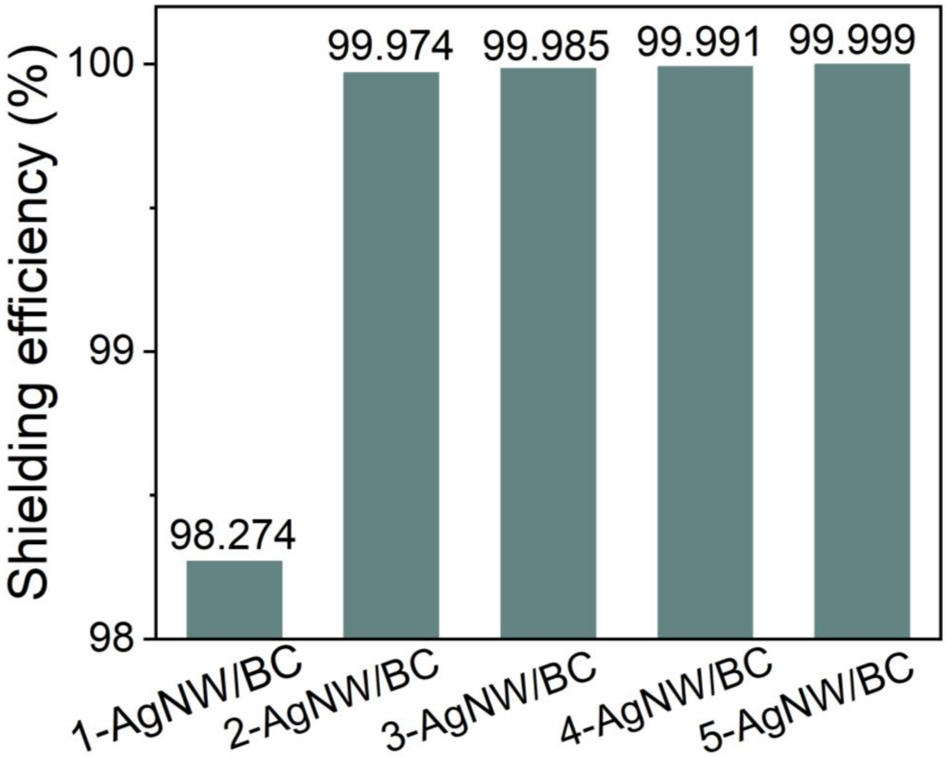

Figure S6. EMI shielding efficiencies of AgNW/BC films.

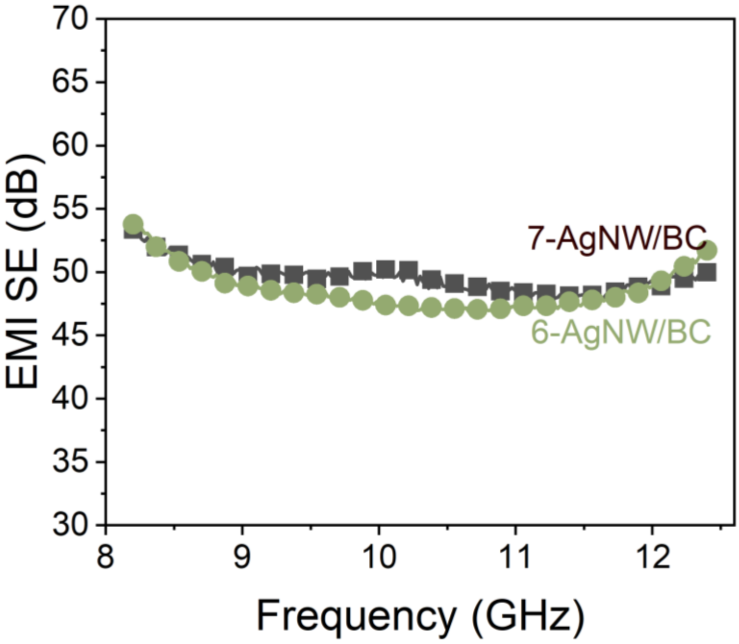

Figure S7. EMI SE of the AgNW/BC films.

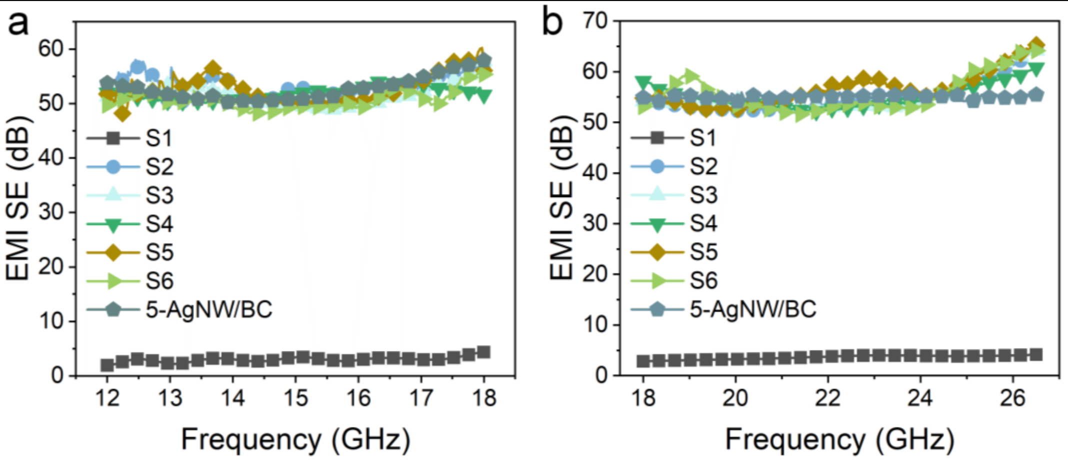

Figure S8. EMI shielding performance in (a) Ku-band and (b) K-band of CNF@BNNS$_{30}$/BC, CNF@BNNS/5-AgNW/BC, and 5-AgNW/BC composite films.

Table S2. The sheet resistance of 6-AgNW/BC and 7-AgNW/BC films.

Sample	Rs (Ω sq^{-1})	Rs (Ω sq^{-1})	Rs (Ω sq^{-1})	Rs (Ω sq^{-1})	Rs (Ω sq^{-1})	Rs (Ω sq^{-1})	Rs (Ω sq^{-1})	Rs (Ω sq^{-1})	Average Rs (Ω sq^{-1})
6-AgNW/BC	0.53	0.48	0.50	0.49	0.47	0.53	0.56	0.47	0.50±0.03
7-AgNW/BC	0.55	0.52	0.51	0.48	0.47	0.45	0.46	0.52	0.50±0.04

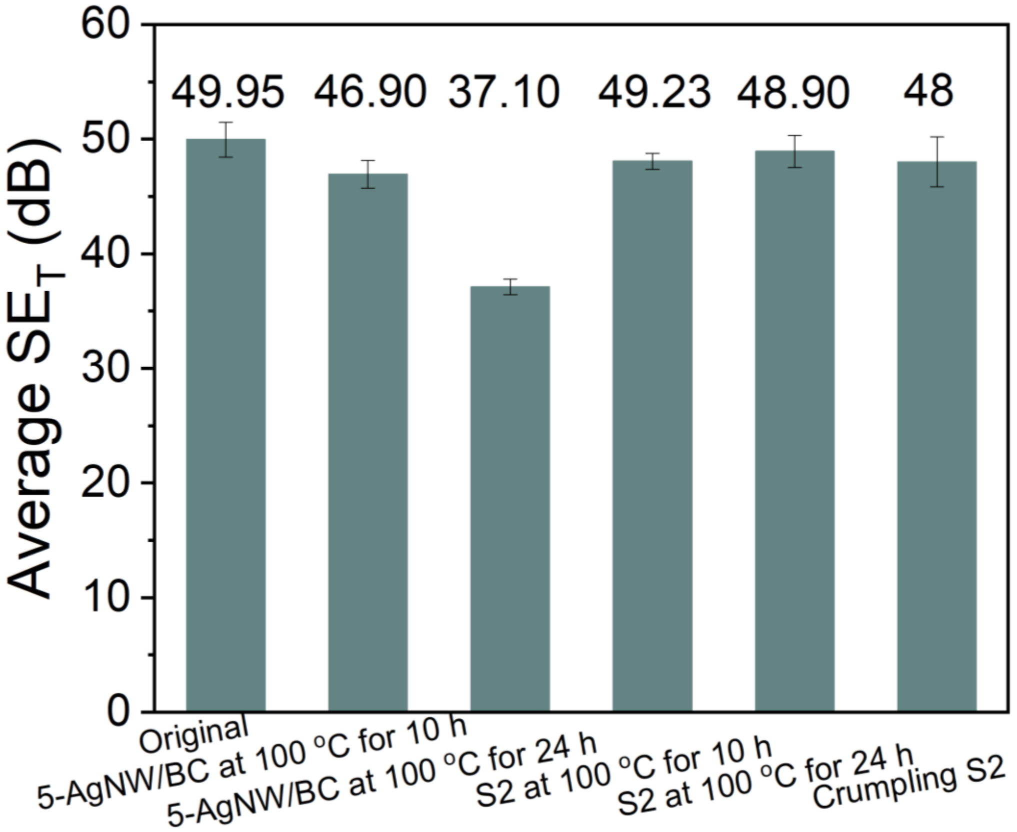

Figure S9. (a) The average SE$_T$ of AgNW/BC, CNF@BNNS/AgNW/BC composite

films at 100 ºC for 10 h and 24 h, and the average SE_T of the crumpling CNF@BNNS/AgNW/BC composite films.

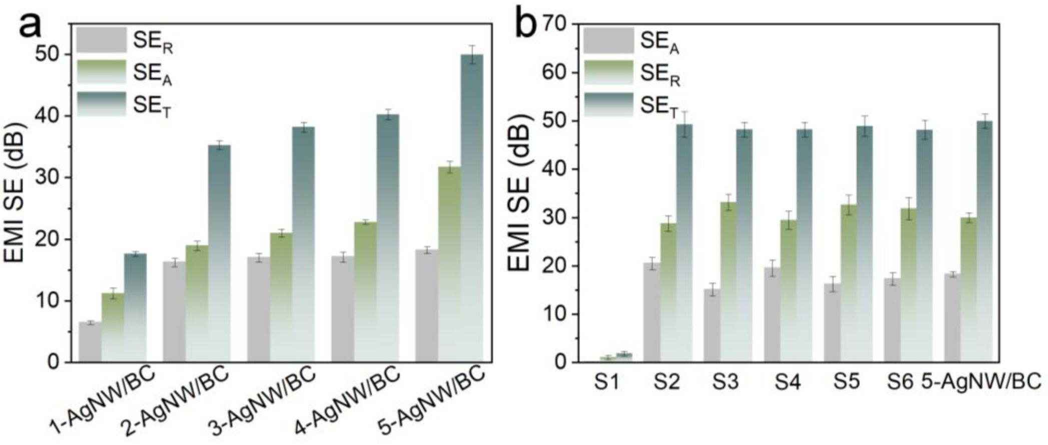

Figure S10. (a) Average SE_R, SE_A, and SE_T values of AgNW/BC films. (b) Average SE_R, SE_A, and SE_T values of 5-AgNW/BC, CNF@BNNS$_{30}$/BC, and CNF@BNNS/AgNW/BC composite films.

Table S3. Comparison of the EMI shielding performance in this work and previously reported composite films.

Materials	Loading (wt%)	EMI SE (dB)	Ref.and year.
GNS/CNF	45	27.40	2020 [3]
GNS	100	50	2018 [4]
GNS/CNF	17.20	18	2016 [5]
CNT@PDA/EVA	70	32.40	2020 [6]
CNTs/BN/rubber	10	20.80	2015 [7]
CNTs/RGO	91	31.20	2020 [4]
CNT/ANF	40	22.70	2022 [8]
MXene/ANF	80	49.50	2018 [9]
Mxene/CNF	90	25.80	2020 [10]
MXene/CNF	50	40	2021 [11]
PVDF/MXene/AgNW	8	25.87	2016 [12]
rGO/AgNW/rGO	35	38	2021 [13]
ANF/AgNW	25	63.30	2016 [14]
CNF@BNNS$_{30}$/5-AgNW/BC	4	48.20	This work

Table S4. Comparison of the EMI shielding performance in this work and previously reported composite films.

Materials	Loading (mg)	EMI SE (dB)	Ref.
GNS/CNF	12.50	27.40	[3]
GNS/Cellulose	300	49.50	[15]
CNT@PDA/EVA	50	32.40	[6]
CNTs/RGO	0.16	31.20	[16]
CNT/ANF	0.68	54.40	[8]
MXene/PLA	12	55.40	[17]
MXene/ANF	80	49.70	[9]
PVDF/MXene/AgNW	100	25.87	[12]
$CoFe_2O_4$@MXene/AgNW/CNF	60	70.90	[18]
ANF/AgNW	1000	63.30	[14]
$AgNW_3$/BC	3	38.18	This work
$AgNW_4$/BC	4	40.21	This work
$AgNW_5$/BC	5	48.20	This work

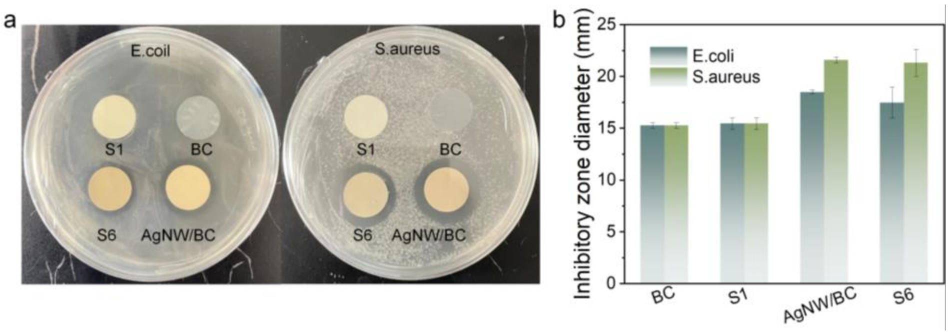

Figure S11. Antibacterial and antibiofilm tests of different samples in E.coil and S.aureus bacterial suspensions. (a) Images and (b) size statistics of inhibitory zone diameter.

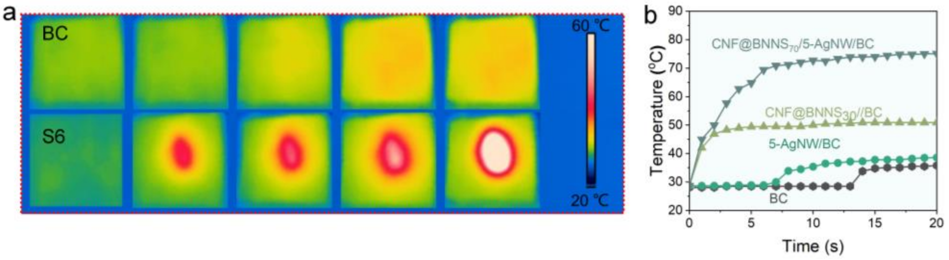

Figure S12. (a) IR images and (b) IR thermal center point temperature curves of BC, 5-AgNW/BC, CNF@$BNNS_{30}$/BC, and CNF@$BNNS_{70}$/5-AgNW/BC composite films.

Table S5. Comparison of thermal conductivity in this work and reported literature.

Composites	Loading	Thermal conductivity (W m^{-1} K^{-1})	Ref.
BN+graphene	50	2.11	[19]
Mxene	20	0.95	[20]
Mxene+FeNi	60	4.72	[21]
AIN	25	4.20	[22]
Mxene+BN	44	4.28	[23]
BN	50	2.58	[24]
AgNW+BNNS	50	8.12	[25]
PDA@GO	0.93	5.60	[26]
Graphene	20	0.59	[27]
BNNS	40	0.67	[28]
BNNS+CNT	70	6.30	[29]
BNNS+AgNW	38.46	0.24	[30]
BNNS+GNS	30	2.53	[31]
BNNS	50	0.62	[32]
BNNS	94	6.90	[33]
Carbon nanotube arrays@ZIF-67	100	9.25	[34]
AgNW+BNNS	52.38	5.02	This work
AgNW+BNNS	61.90	6.46	This work
AgNW+BNNS	71.42	8.85	This work

Table S6. Comparison of comprehensive performance in this work and reported literature.

Material	EMI shielding (dB)	Anti-biofouling (mm)	Joule heating (°C)	Thermal conductivity (W m^{-1} K^{-1})	Sheet resistance (Ω sq^{-1})	Response time (s)	Ref.
GNS/PTFE	50.85		70.5	2.92		150	[35]
Si-Mxene/CNF	39.50		44				[36]
MXene@PEG/ $Mg(OH)_2$	38.80			0.95			[20]
Janus (BNNS/ANF)-(AgNWs/ANF)			148	8.12		10	[25]
AgNW/CS	53.80	3.50					[37]
PVA/Mxene/ AgNW	25.87			0.78			[12]
Fe_3O_4/$Ti_3C_2T_x$/ PVA	40			2.86			[38]
MXene/PDMS/BN	35.20			0.65			[39]
CNM/PDA/ Ag/PDMS		1			0.20		[40]
ANF-1500	41.40				1.0		[41]
Mxene/ANF	44		70	0.43		65	[42]
PVDF/Mxene	42.90		72		2.74	54	[43]
NR-CNT	32.40		104.90	6.97			[44]
PMCNT	45.70		101.40	1.95			[45]
$MoSe_2$@MXene/ CF	37		80		12	25	[46]
This work	48.60	20	196	8.85	0.52	8	

References

[1] Z. Li, Z. Yu, Y. Wu, X. Wu, Y. Wan, Y. Yuan, N. Wang, *Chem. Eng. J.* **2020**, *390*, 124648.

[2] Y. Yong, G. Dykes, S. Lee, W. Choo, *J. Appl. Microbiol.* **2019**, *126*, 68.

[3] L. Li, Z. Ma, P. Xu, B. Zhou, Q. Li, J. Ma, C. He, Y. Feng, C. Liu, *Part A Appl. Sci. Manuf.* **2020**, *139*, 106134.

[4] E. Zhou, J. Xi, Y. Guo, Y. Liu, Z. Xu, L. Peng, W. Gao, J. Ying, Z. Chen, C. Gao, *Carbon* **2018**, *133*, 316.

[5] Y. Yao, X. Zeng, G. Pan, J. Sun, J. Hu, Y. Huang, R. Sun, J. B. Xu, C. P. Wong, *ACS Appl. Mater. Interfaces* **2016**, *8*, 31248.

[6] Z. Wang, Y. Yang, Z. Zheng, R. Lan, K. Dai, L. Xu, H. Huang, J. Tang, J. Xu, Z. Li, *Compos. Sci. Technol.* **2020**, *194*, 108190.

[7] H. Huang, C. Liu, D. Zhou, X. Jiang, G. Zhong, D. Yan, Z. Li, *J. Mater. Chem. A* **2015**, *3*, 4983.

[8] P. Hu, J. Lyu, C. Fu, W.-b. Gong, J. Liao, W. Lu, Y. Chen, X. Zhang, *ACS Nano* **2020**, *14*, 688.

[9] J. Yao, L. Zhang, F. Yang, Z. Jiao, X. Tao, Z. Yao, Y. Zheng, J. Zhou, *Chem. Eng. J.* **2022**, *446*, 136945.

[10] W.-T. Cao, F.-F. Chen, Y.-J. Zhu, Y.-G. Zhang, Y.-Y. Jiang, M.-G. Ma, F. Chen, *ACS Nano* **2018**, *12*, 4583.

[11] B. Zhou, Z. Zhang, Y. Li, G. Han, Y. Feng, B. Wang, D. Zhang, J. Ma, C. Liu, *ACS Appl. Mater. Interfaces* **2020**, *12*, 4895.

[12] H. Cheng, Y. Pan, Q. Chen, R. Che, G. Zheng, C. Liu, C. Shen, X. Liu, Adv. Compos. *Hybrid Ma.* **2021**, *4*, 505.

[13] P. Kumar, F. Shahzad, S. Hong, C. Koo, *RSC Adv.* **2016**, *6*, 101283.

[14] S. Li, K. Qian, S. Thaiboonrod, H. Wu, S. Cao, M. Miao, L. Shi, X. Feng, *Part A Appl. Sci. Manuf.* **2021**, *151*, 106643.

[15] Z. Guo, P. Ren, Z. Dai, Z. Zong, F. Zhang, Y. Jin, F. Ren, *Cellulose* **2021**, *28*, 3135.

[16] L. Kong, X. Yin, H. Xu, X. Yuan, T. Wang, Z. Xu, J. Huang, R. Yang, H. Fan, *Carbon* **2019**, *145*, 61.

[17] Z. Du, K. Chen, Y. Zhang, Y. Wang, P. He, H. Mi, Y. Wang, C. Liu, C. Shen, *Commun.* **2021**, *26,* 100770.

[18] Z. Guo, P. Ren, Z. Lu, K. Hui, J. Yang, Z. Zhang, Z. Chen, Y. Jin, F. Ren, *ACS Appl. Mater. Interfaces* **2022**, *14*, 41468.

[19] M. Tsai, I. Tseng, J. Chiang, J. Li, *ACS Appl. Mater. Interfaces* **2014**, *6*, 8639.

[20] D. Xu, Q. Huang, L. Yang, Y. Chen, Z. Lu, H. Liu, P. Han, L. Guo, C. Wang, C. Liu, *Carbon* **2023**, *202*, 1.

[21] B. Zhao, Z. Ma, Y. Sun, Y. Han, J. Gu, *Small Struct*. **2022**, *3*, 2200162.

[22] K. Zhang, P. Tao, Y. Zhang, X. Liao, S. Nie, *Carbohydr. Polym*. **2019**, *213*, 228.

[23] S. Lee, J. Kim, *Polymer*. **2021**, *13*, 379.

[24] X. Wang, M. Sun, R. Wang, L. Jiao, H. Bian, H. Dai, *Mater. Design* **2022**, *214*, 110379.

[25] Y. Han, K. Ruan, J. Gu, *Nano Res*. **2022**, *15*, 4747.

[26] Y. Wang, Z. Zhang, T. Li, P. Ma, X. Zhang, B. Xia, M. Chen, M. Du, T. Liu, W. Dong, *ACS Appl. Mater. Interfaces* **2020**, *12*, 44273.

[27] H. Guo, X. Li, B. Li, J. Wang, S. Wang, *Mater. Design* **2017,** *114*, 355.

[28] S. Zuo, Y. Lan, J. Luo, F. Zhou, L. Xu, S. Xie, X. Wei, L. Zhou, L. Ma, X. Li, C. Yin, *ACS Omega* **2022**, *7*, 3273.

[29] L. Yang, L. Zhang, C. Li, *Compos. Sci. Tech*. **2020**, *200*, 108429.

[30] J. Huang, L. Hua, L. Song, J. Li, S. E, Z. Lu, *Mater. Today Sustain*. **2023**, *21*, 100307.

[31] D. Liu, H. Chi, C. Ma, M. Song, P. Zhang, P. Dai, *Compos. Sci. Tech*. **2022**, *220*, 109292.

[32] M. Lin, Y. Li, K. Xu, Y. Ou, L. Su, X. Feng, J. Li, H. Qi, D. Liu, *Compos. Sci. Tech*. **2019**, *175*, 85.

[33] X. Zeng, L. Ye, S. Yu, H. Li, R. Sun, J. Xu, C. Wong, *Nanoscale* **2015**, *7*, 6774.

[34] Y. Wang, Y. Yang, M. Miao, X. Feng, *Mat. Today Phys.* **2023**, *35*, 101110.

[35] Q. Wei, L. Li, Z. Deng, G. Wan, Y. Zhang, C. Du, Y. Su, G. Wang, *Small* **2023,** *19*, 2302082.

[36] W. Xin, M. Ma, F. Chen, *ACS Appl. Nano Mater*. **2021**, *4*, 7234.

[37] M. Zhu, X. Yan, Y. Lei, J. Guo, Y. Xu, H. Xu, L. Dai, L. Kong, *ACS Appl. Mater. Interfaces* **2022**, *14*, 14520.

[38] Y. Zhang, K. Ruan, J. Gu, *Small* **2021**, *17*, 2101951.

[39] H. Liu, R. Fu, X. Su, B. Wu, H. Wang, Y. Xu, X. Liu, *Compos. Commun*. **2021**, *23*, 100593.

[40] J. Li, K. Luo, J. Zhang, J. Lei, H. Lin, J. Tang, G. Zhong, D. Yan, Z. Li, *Chem. Eng. J.* **2022**, *439*, 135662.

[41] B. Zhou, G. Han, Z. Zhang, Z. Li, Y. Feng, J. Ma, C. Liu, C. Shen, *Carbon* **2021**, *184*, 562.

[42] C. Liu, Y. Ma, Y. Xie, J. Zou, H. Wu, S. Peng, W. Qian, D. He, X. Zhang, B. Li, C. Nan, *ACS Appl. Mater. Interfaces* **2023**, *15*, 4516.

[43] Y. Li, B. Zhou, Y. Shen, C. He, B. Wang, C. Liu, Y. Feng, C. Shen, *Compos. B: Eng.* **2021**, *217*, 108902.

[44] M. Fan, S. Li, L. Wu, L. Li, M. Qu, J. Nie, R. Zhang, P. Tang, Y. Bin, *Chem. Eng. J.* **2022**, *433*, 133499.

[45] J. Li, Y. Wang, T. Yue, Y. Gao, Y. Shi, J. Shen, H. Wu, M. Wang, *Compos. Sci. Tech.* **2021**, *206*, 108681.

[46] J. Xie, Y. Zhang, J. Dai, Z. Xie, J. Xue, K. Dai, F. Zhang, D. Liu, J. Cheng, F. Kang, B. Li, Y. Zhao, L. Lin, Q. Zheng, *Small* **2023**, *19*, 2205853.
